# Modulatory effect of camel milk on intestinal microbiota of mice with non-alcoholic fatty liver disease

**DOI:** 10.3389/fnut.2022.1072133

**Published:** 2022-12-01

**Authors:** Shiqi Hao, Liang Ming, Yafei Li, Haodi Lv, Lin Li, Tuyatsetseg Jambal, Rimutu Ji

**Affiliations:** ^1^Key Laboratory of Dairy Biotechnology and Engineering, Ministry of Education, Inner Mongolia Agricultural University, Hohhot, China; ^2^China-Mongolia Joint Laboratory for Biomacromolecule Research, Ulaanbaatar, Mongolia

**Keywords:** non-alcoholic fatty liver disease, camel milk, intestinal flora, glucolipid metabolism, regulating function

## Abstract

Non-alcoholic fatty liver disease (NAFLD) is a common metabolic disease of life, usually caused by unhealthy diet and lifestyle. Compared to normal individuals, the structure of the intestinal flora of NAFLD patients is altered accordingly. This study investigates the effect of camel milk on the regulation of intestinal flora structure in mice with high-fat diet-induced NAFLD. NAFLD model was established by feeding C57BL/6J mice a high-fat diet for 12 weeks, meanwhile camel milk (3.0 g/kg/d), cow milk (3.0 g/kg/d), and silymarin (200 mg/kg/d) were administered by gavage, respectively. Food intake and changes of physiological indexes in mice were observed and recorded. The 16S rRNA gene V3-V4 region was sequenced and the intestinal flora diversity and gene function were predicted in the colon contents of mice from different group. The results showed that camel milk enhanced glucolipid metabolism by downregulate the levels of blood glucose and triglyceride (TG) in serum, reduced lipid accumulation by downregulate the level of TG in the liver and improved liver tissue structure in NAFLD mice (*p* < 0.05). Meanwhile, camel milk had a positive modulatory effect on the intestinal flora of NAFLD mice, increasing the relative abundance of beneficial bacteria and decreasing the relative abundance of harmful bacteria in the intestinal flora of NAFLD mice, and silymarin had a similar modulatory effect. At the genus level, camel milk increased the relative abundance of *Bacteroides*, norank_f_*Muribaculaceae* and *Alloprevotella* and decreased the relative abundance of *Dubosiella* and *Coriobacteriaceae_UCG-002* (*p* < 0.05). Camel milk also enhanced Carbohydrate metabolism, Amino acid metabolism, Energy metabolism, Metabolism of cofactors and vitamins and Lipid metabolism in NAFLD mice, thus reducing the degree of hepatic lipid accumulation in NAFLD mice and maintaining the normal structure of the liver. In conclusion, camel milk can improve the structure and diversity of intestinal flora and enhance the levels of substance and energy metabolism in NAFLD mice, which has a positive effect on alleviating NAFLD and improving the structure of intestinal flora.

## Introduction

Non-alcoholic fatty liver disease (NAFLD), a major community health challenge for the World Health Organization, is one of the most common and preventable liver diseases, affecting approximately one-quarter of the population, and is expected to become a worldwide epidemic in the future (1, 2). According to the severity of liver disease, NAFLD is classified as simple hepatic lipid accumulation, hepatic steatosis, inflammatory infiltration of liver cells, liver fibrosis, cirrhosis, and hepatocellular carcinoma. Currently, some studies also called hepatic lipid accumulation and hepatic steatosis as metabolic associated fatty liver disease (MAFLD) (3). NAFLD occurs for a variety of reasons, including but not limited to unhealthy eating habits, obesity, physical or psychological stress, and insulin resistance; and the development of NAFLD is a risk signal for the development of diabetes, atherosclerosis, and other metabolic syndromes (4–7). Generally, a healthy person can convert sugar and fat into energy through digestion, absorption, and metabolism or store them as glycogen and lipid droplets through liver metabolism. However, the liver will convert a large amount of blood glucose and triglycerides that cannot be consumed into lipid droplets and store them in fat cells because of a lack enough of exercise or consuming a high-fat and high-sugar diet for a long time. Firstly, lipid droplets accumulate in the liver, and the liver’s function of glycolipid metabolism will be impaired, and then lipid droplets will accumulate in the waist and abdomen, causing non-alcoholic fatty liver and obesity. Overeating in obese people causes the body to resist insulin due to sudden rise and drop in blood glucose (6). Due to the body’s resistance to insulin, high blood glucose increases the burden of liver glucolipid metabolism which damages the liver. In addition, the “double hit” theory and the “multiple hit” theory are also leading to the occurrence of NAFLD. “Double hit” means that NAFLD is caused by hepatic steatosis and a subsequent systemic inflammatory response (8–10). The “multiple-hit” theory is an extension of the “double-hit” theory, which states that NAFLD is caused by impairments of physiological functions, including insulin resistance, lipotoxicity, oxidative damage, endoplasmic reticulum stress, mitochondrial dysfunction, adipose tissue dysfunction, innate immune dysregulation, and cytokine secretion (11, 12). Improving dietary structure, increasing exercise, enhancing body immunity, clinical medication, cold stimulation, and local hyperthermia therapy were considered to prevent and treat NAFLD. However, such methods have certain limitations, and clinical medications generally have certain nephrotoxicity, so the use of functional foods (Functional foods are foods with specific nutritional health functions, which are suitable for consumption by specific groups of people and have the function of regulating the body, not for the purpose of treatment) to alleviate liver lipid deposition and adjuvant treatment of NAFLD is necessary (13, 14).

Camel milk, known as “desert platinum” because of its minimal production, is a nutritionally balanced food with various biological functions such as anti-allergy, antioxidant, and antihypertensive (15). Because camel milk is rich in immunoglobulins IgG, IgA, and various vitamins (vitamin A, vitamin B2, vitamin C, and vitamin E), lower in fatty acids and cholesterol, and lacking in allergens because of the absence of beta-lactoglobulin, camel milk is often used in arid regions of Asia and Africa as a treatment for asthma, edema, and diabetes (15–18). In addition, camel milk protects the kidney from oxidative stress by Nrf2/HO-1 and AKT/eNOS/NO pathway and maintains kidney function. Several researches have demonstrated that camel milk significantly alleviate alcoholic liver disease by activating the IL-17 and TNF pathways (19–23). Type II diabetes is a typical metabolic disorder with significant insulin resistance and unstable blood glucose before the onset of the disease. Camel milk effectively regulated blood glucose and availably improved the body’s insulin resistance and prevented type II diabetes (24, 25).

The intestinal flora, a complex ecosystem, is closely linked to the health of the body. The intestinal flora and its metabolites (such as amino acids, nucleotides, polysaccharides, lipids, vitamins, antibiotics, toxins, hormones) are important factors affecting the health of the body, and changes in the structure of the intestinal flora often mean the onset of disease (26, 27). In the process of establishing a diarrhea model, Bao et al. (28) found that the intestinal flora structure of mice changes over time, with the relative abundance of beneficial bacteria decreasing and the relative abundance of harmful bacteria increasing. Treatment of obesity and type II diabetes by improving intestinal flora has been reported (29–31). Camel milk has also been reported to alter intestinal flora in mice, which increased the relative abundance of *Allobaculum* and *Akkermansia* and a decreased *Erysipelotrichaceae* and *Desulfovibrionaceae* (32). Therefore, camel milk can be considered to be used to regulate the intestinal flora of NAFLD mice and improve the metabolic status of the body, alleviating NAFLD.

In this study, NAFLD model was established by feeding C57BL/6J mice with high-fat diet for 12 weeks. Meanwhile, camel milk, cow milk, and silymarin were gavaged daily to mice in camel milk group, cow milk group and silymarin group, respectively. The changes of the physiological and biochemical indexes of NAFLD mice were observed to study the effects of camel milk, cow milk and silymarin on the alleviation of NAFLD and the intestinal flora of mice.

## Materials and methods

### Experimental reagents and preparation of camel milk

Total triglycerides (TG) kit (Item No. A110-1-1) and Total cholesterol (TC) kit (Item No. A111-1-1) were purchased from Nanjing Jiancheng Institute of Biological Engineering. ELISA kit obtained from Shanghai EnzymeLink Biotechnology Co. Silymarin and carboxymethyl cellulose were purchased from Shanghai Yuanye Biological Co.

Camel milk and cow milk were collected from the pasture area of Alax Right Banner, Alax League, Inner Mongolia, China. Fresh camel milk was transported back to the laboratory after quick-freezing in liquid nitrogen, and stored at −80°C. Frozen camel milk was thawed in cold water, centrifuged (4°C, 5000 r min^–1^, 30 min) to separate the fat, freeze-dried, sieved and packaged, and stored at 4°C. High-fat feed (45% fat, 17% fructose, 1.25% cholesterol, No. TP32003) was purchased from Nantong TROPHY Feed Technology Co. Camel milk, cow milk and silymarin were mixed with 0.5% carboxymethylcellulose (CMC), respectively.

### Animal ethics statement

Fifty SPF-grade male C57BL/6J mice were purchased from SPF (Beijing) Biotechnology Co., Ltd. Animals were housed in IVC animal experimental system with a housing temperature of 20°C ± 1°C, relative humidity of 50% ± 5%, and a 12-h day/night cycle. Mice were acclimatized and fed for 7 days prior to the start of the test, with free access to food and sterile distilled water. The control diet was gradually replaced with high-fat diet during acclimatization feeding. All animal experiments are conducted in strict accordance with the guidelines for the care and use of laboratory animals as described by the National Institutes of Health, minimizing the amount of mice and reducing their suffering while achieving the experimental objectives. The experimental program was approved by the Ministry of Education Key Laboratory of Dairy Biotechnology and Engineering, Inner Mongolia Agricultural University (License No.: SYXK Meng 2020-0002). During the experimental period, the NC group was fed the control diet, and the other groups were fed high-fat feed.

### Experimental grouping and establishment of non-alcoholic fatty liver disease mice model

After 7 days of acclimatization feeding, fifty mice were randomly divided into negative control group (NC group, *n* = 10), model group (Mod group, *n* = 10), camel milk group (CaM group, *n* = 10), cow milk group (CoM group, *n* = 10), and positive control group (PC group, *n* = 10). After 12 weeks, the NAFLD model that was characterized by increased liver index and the levels of TG and TC in the liver was established. To alleviate NAFLD in mice, camel milk, cow milk and silymarin were administered by gavage. Twice daily (9:00 a.m and 4:00 p.m), CaM group was gavaged with camel milk (3.0 g/kg/d), CoM group was gavaged with cow milk (3.0 g/kg/d), PC group was gavaged with silymarin (200 mg/kg/d), Mod group and NC group were gavaged with 0.5% CMC (0.2 ml/d/per) (Table 1). The oral glucose tolerance test (OGTT) and insulin tolerance test (ITT) were measured at 13 weeks in each group of mice with fasting without water for 12 h. The mice were anesthetized with 3% pentobarbital sodium (45 mg/kg) and sacrificed by severing the neck. Blood sample, liver tissues and colon contents were collected. Serum was isolated from blood samples by centrifugation at 3,000 r/min, for 20 min at 4°C. The liver was used for the preparation of HE-stained sections to observe pathological structure. The levels of TG and TC in serum and liver were measured and the diversity of intestinal flora was analyzed, respectively.

**TABLE 1:** Animal grouping and handing.

Groups	Feed types	Gastric perfusion scheme	Time	Period
NC	Control diet	0.5% CMC 0.2 ml/d	9:00 a.m. and 4:00 p.m.	12 weeks
Mod	High fat diet	0.5% CMC 0.2 ml/d	9:00 a.m. and 4:00 p.m.	12 weeks
CaM	High fat diet	Camel milk 3.0 g/kg/d	9:00 a.m. and 4:00 p.m.	12 weeks
CoM	High fat diet	Cow milk 3.0 g/kg/d	9:00 a.m. and 4:00 p.m.	12 weeks
PC	High fat diet	Silymarin 200 mg/kg/d	9:00 a.m. and 4:00 p.m.	12 weeks

NC, negative group; Mod, model group; CaM, camel milk group; CoM, cow milk group; PC, positive group.

### Detection of physiological indexes and observation of animal phenotype

Body weight, blood glucose, liver index, and the levels of TG and TC in the liver are important indices. Body weight and blood glucose of mice were measured weekly, blood glucose was detected using a blood glucose meter. Feed consumption was weighed and recorded every 3 days. The liver was weighed after dissecting the mice and the liver index was calculated. Liver was used to determine TG level and TC level and to make pathological sections (hematoxylin eosin staining) for observing the pathological structure of the liver. Liver tissue samples stored in 4% paraformaldehyde were gradient dehydrated and paraffin-embedded, and then cut into 5-μm sections. The sections were stained with hematoxylin staining solution and eosin staining solution, dried and placed under a microscope to observe the liver tissue structure. Liver index is calculated as follows.


$$L\,i\,v\,e\,r\,i\,n\,d\,e\,x=\frac{F\,r\,e\,s\,h\,w\,e\,i\,g\,h\,t\,o\,f\,l\,i\,v\,e\,r\,\left(g\right)}{b\,o\,d\,y\,w\,i\,g\,h\,t\,\left(g\right)}\times 100\%$$

### Detection of biochemical indexes

The levels of alanine transaminase (ALT), aspartate aminotransferase (AST), leptin (LEP), adiponectin (ADPN), high density lipoprotein cholesterol (HDL-c), low density lipoprotein cholesterol (LDL-c), interleukin (IL-6), tumor necrosis factor-alpha (TNF-alpha), and insulin (INS) in serum were measured according to manufacturer’s instructions by ELISA kits (Item number are ml063179, ml058577, ml002287, ml057809, ml037765, ml037825, ml063159, ml002095, ml001983, respectively). And the levels of total triglycerides (TG) and total cholesterol (TC) in serum and liver were detected, respectively.

After 12 weeks of gavage, mice were fasted without water for 12 h and gavaged with 30% glucose solution (1.5 g/kg), then the blood glucose values were measured at 0, 30, 60, 90, and 120 min. Mice were given intraperitoneal injection of insulin (0.5 U/kg), and the blood glucose values at 0, 30, 60, 90, and 120 min were detected. While the area under the curve of ITT and OGTT were calculated. According to the formulas, the insulin resistance index (HOMA-IRI) and insulin sensitivity index (HOMA-ISI) were calculated (33).


$$H\,O\,M\,A-I\,R\,I=F\,B\,G\times \frac{F\,i\,n\,s}{22.5}$$

$$H\,O\,M\,A-I\,S\,I=l\,n\,\left(\frac{1}{F\,B\,G}\times F\,i\,n\,s\right)$$

*Fins* is mice fasting insulin, *FBG* is mice fasting blood glucose.

### DNA extraction and PCR amplification

To observe the differences in intestinal flora different groups of mice, fresh fecal was collected in lyophilization tubes and quick-frozen in liquid nitrogen during week 12, and DNA of mouse intestinal flora was extracted using the E.Z.N.A.Cycle-Pure Kit (Omega BIO-TEK, Norcross, GA, USA) (Table 2). DNA integrity was detected by 1% agarose gel electrophoresis. The DNA purity and concentration were assayed by NanoDrop2000 (Thermo Scientific, Wilmington, NC, USA). The V3-V4 variable region of bacterial 16S rRNA were amplified by an ABI GeneAmp 9700 PCR thermocycler (ABI, CA, USA). The PCR amplification of 16S rRNA genes was performed as follows: initial denaturation at 95°C for 3 min, followed by 30 cycles of denaturing at 95°C for 30 s, annealing at 55°C for 30 s, extension at 72°C for 8 min, and end at 4°C. Quantitative analysis was performed using PCR after amplification (Table 3). PCR amplification results were detected by 2% agarose gel electrophoresis, purified using AxyPre DNA Gel Extraction Kit (Axygen Biosciences, Union City, CA, USA), and quantified using Quantus™ Fluorometer (Promega, Madison, WI, USA). Primer information and PCR reaction conditions are listed below.

**TABLE 2:** Primer sequence.

Primer	Sequence
338F	5′-ACTCCTACGGGAGGCAGCAG-3′
806R	5′-GGACTACHVGGGTW TCTAAT-3′

**TABLE 3:** PCR amplification system.

System	Volume
5 × FastPfu buffer	4 μl
2.5 mm dNTPs	2 μl
Forward primer (5 μm)	0.8 μl
Reverse primer (5 μm)	0.8 μl
FastPfu polymerase	0.4 μl
BSA	0.2 μl
Template DNA	10 ng
ddH_2_O	Add to 20 μl

### Illumina MiSeq sequencing

Purified amplicons were pooled in equimolar and paired-end sequenced on an Illumina MiSeq PE300 platform/NovaSeq PE250 platform (Illumina, San Diego, CA, United States) according to the standard protocols by Majorbio Bio-Pharm Technology Co. Ltd. (Shanghai, China). The raw reads were deposited into the National Center of Biotechnology Information (NCBI) Sequence Read Archive (SRA) database (accession number: PRJNA889750).

### Processing of sequencing data

The original 16S rRNA was isolated screened and combined using the method of Bao et al. (28). The specific conditions were as follows: (i) the 300 bp reads were truncated at any site receiving an average quality score of <20 over a 50 bp sliding window, the truncated reads shorter than 50 bp were discarded, and reads containing ambiguous characters were also removed; (ii) only overlapping sequences longer than 10 bp were assembled according to their overlapped sequences; the maximum mismatch ratio of overlap region is 0.2; reads that could not be assembled were discarded; (iii) samples were distinguished according to the barcode and primers, and the sequence direction was adjusted, exact barcode matching, 2 nucleotide mismatch in primer matching.

Operational taxonomic units (OTUs) with 97% similarity cutoff were clustered using UPARSE version 7.1, and chimeric sequences were identified and removed. The taxonomy of each OTU representative sequence was analyzed by RDP Classifier version 2.2 against the 16S rRNA database (e.g., Silva v138) using a confidence threshold of 0.7.

### Statistics and analysis of data

Mothur software (v.1.30.2) was used to calculate the alpha diversity index of OTU level, such as Ace, Chao, and Shannon index, and draw the dilution curve. Beta diversity distance matrix was calculated by Qiime software, and principal co-ordinates analysis (PCoA) and non-metric multidimensional scaling (NMDS) diagrams were drawn, respectively. Through linear discriminant analysis effect size (LEfSe) method, the species with significant differences in abundance among different groups were found, and the final difference species were obtained by linear discriminant analysis (LDA). The gene function was predicted and analyzed by PICRUSt2 software, and the relative abundance heat map of functional modules was drawn to find out KEGG metabolic pathway involved by intestinal microbes. All the above drawings were made with R language tools.

The experimental data were expressed as mean ± SD. SPSS 23 software was used for statistical analysis, and Graphpad prism 9.0 was used for drawing. After one-way analysis of variance (ANOVA), statistical analysis between groups was performed by Duncan’s multiple range tests, and the differences were considered as significance at *p* < 0.05. Bioinformatic analysis was carried out on Majorbio I-Sanger cloud platform.

## Results

### Establishment of non-alcoholic fatty liver disease mouse model and the effect of camel milk on the physiology of non-alcoholic fatty liver disease mice

**FIGURE 1:**
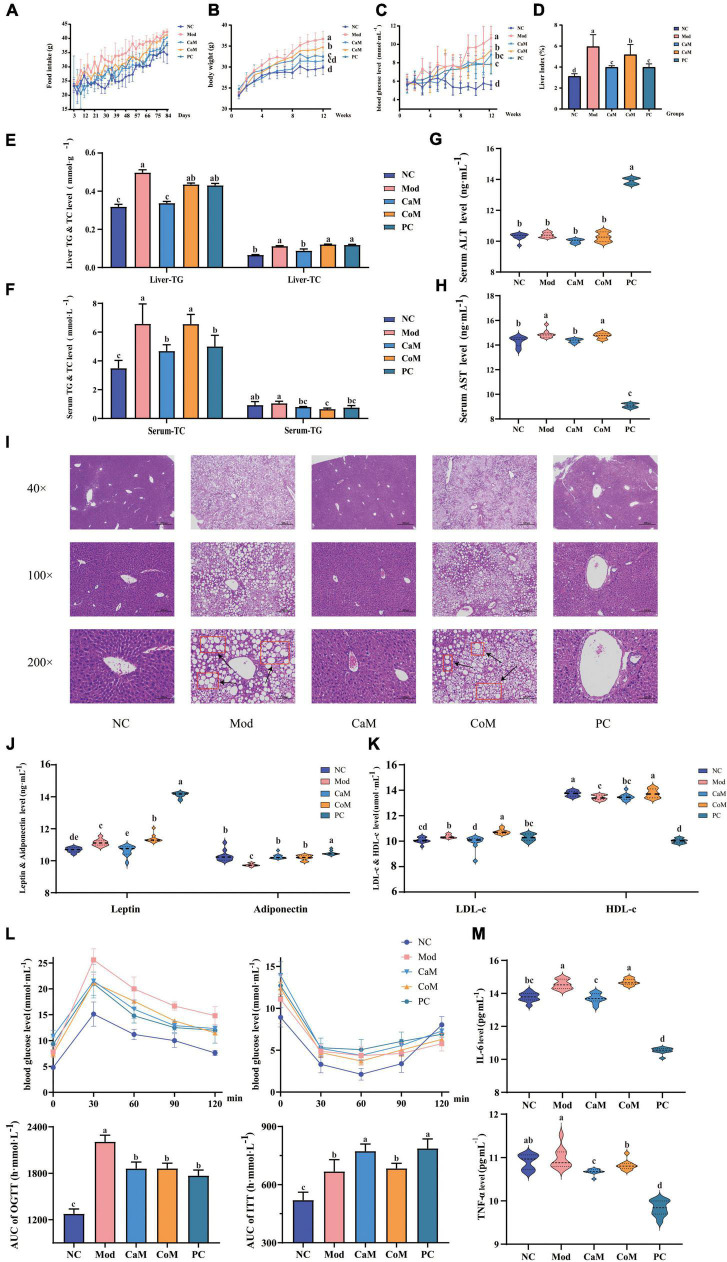
Physiological indicators and serum biochemical indicators: **(A)** Food intake. **(B)** Body weight. **(C)** Blood glucose. **(D)** Liver index. **(E)** The levels of triglyceride (TG) and total cholesterol (TC) in liver. **(F)** The levels of TG and TC in serum. **(G)** Serum alanine transaminase (ALT) level. **(H)** Serum aminotransferase (AST) level; **(I)** Liver pathology section. **(J)** The levels of leptin and adiponectin in serum. **(K)** The levels of low density lipoprotein cholesterol (LDL-c) and high density lipoprotein cholesterol (HDL-c) in serum; oral glucose tolerance test (OGTT) and insulin tolerance test (ITT) **(L)**. **(M)** The levels of interleukin (IL-6) and tumor necrosis factor-alpha (TNF-α) in serum. The experimental results are expressed as the mean ± SD. *n* = 10.

Mice fed high-fat diet for 12 weeks to establish NAFLD model. As shown in Figures 1A–C, at the beginning of the experiment, there were no significant differences in food intake, body weight, and blood glucose between the groups of mice (*p* > 0.05). With the progress of the experiment, the food intake, body weight, and blood glucose of the mice in each group showed an increasing trend. The upward trend of mice in the Mod group was higher than all other groups. At week 12 (Table 4), mice in the Mod group had significantly higher body weight and blood glucose than all other groups (*p* < 0.05).

**TABLE 4:** Body weight and blood glucose at 12 weeks.

Index	NC	Mod	CaM	CoM	PC
Body weight (g)	29.48 ± 1.41^d^	36.88 ± 1.60^a^	31.45 ± 1.27^c^	35.20 ± 1.11^b^	30.84 ± 1.87^c^
Blood glucose (mmol ml^–1^)	5.57 ± 0.46^d^	10.69 ± 1.27^a^	8.85 ± 0.90^b^	9.36 ± 1.68^b^	7.82 ± 1.03^c^

Different superscript letters represent two data with significant differences (*p* < 0.05).

Non-alcoholic fatty liver disease is characterized by abnormally large liver, excessive accumulation of TG in the liver, hepatic steatosis, and structural damage to liver tissue. At week 12 (Figures 1D–F), the liver index and the levels of TG and TC in the liver of mice in the Mod group were notably higher than that of the NC group (*p* < 0.05). The TC level in serum in Mod group was significantly higher comparing with NC group (*p* < 0.05). However, there were not significantly difference between Mod group and NC group in serum TG level (*p* > 0.05, Figures 1E,F). In CaM group, the levels of TG and TC were reduced significantly in the serum and the liver (*p* < 0.05, Figures 1E,F).

The levels of AST and ALT in serum are important indicators to determine the extent of NAFLD development in mice. In our study (Figures 1G,H), there was no significant difference in ALT levels between the NC and Mod groups (*p* > 0.05), while AST levels in the Mod group were significantly higher than those in the NC group (*p* < 0.05). In addition, it can be observed from the histological sections of liver pathology that compared to the NC group (dense structure and neatly arranged hepatocytes), the Mod group of mice had severe lipid accumulation in the liver, the tissue structure was severely disrupted, and the hepatocytes were not arranged in a specific order (Figure 1I). After intervention of camel milk, the level of AST was decreased significantly in serum of mice (*p* < 0.05).

Leptin, adiponectin, HDL-c, and LDL-c are important indicators of lipid metabolism in the body. Compared with the NC group, the levels of leptin and LDL-c in serum were significantly increased and the levels of adiponectin and HDL-c were significantly decreased in the Mod group (*p* < 0.05). However, the levels of leptin and LDL-c were significantly lower and adiponectin in serum was significantly higher in the CaM group compared with Mod group (*p* < 0.05). There was not significantly difference in the serum HDL-c level between CaM group and Mod group (*p* > 0.05, Figures 1J,K).

Glucose tolerance, insulin tolerance, insulin sensitivity, and insulin resistance index respond to the glucose metabolism ability of the mice (Table 5). As shown in Figure 1L, the insulin tolerance of mice in the Mod group was severely impaired, and the blood glucose values of mice in the Mod group were significantly higher than mice in the NC group at 0, 30, 60, and 90 min (*p* < 0.05). Contrary to expectations, it was found that camel milk and silymarin interventions did not reduce the degree of impairment of insulin tolerance in mice by calculating the area under the ITT curve (Figure 1L). Figure 1L showed that the glucose tolerance of the mice in the Mod group suffered impairment, and the blood glucose values of the mice in the Mod group were significantly higher than other groups at 30, 60, 90, and 120 min (*p* < 0.05). And camel milk and silymarin regulated the glucose tolerance of the mice to different degrees (Figure 1L).

**TABLE 5:** Insulin resistance index and insulin sensitivity index of each group.

Groups	HOMA-IRI	HOMA-ISI
NC	2.517 ± 0.201^c^	−4.034 ± 0.079^a^
Mod	5.067 ± 0.725^a^	−4.727 ± 0.146^c^
CaM	3.846 ± 0.391^b^	−4.456 ± 0.105^b^
CoM	4.257 ± 0.748^b^	−4.548 ± 0.173^b^
PC	5.056 ± 0.717^a^	−4.726 ± 0.135^c^

Different superscript letters represent two data with significant differences (*p* < 0.05).

In addition, the inflammation indicators of IL-6 and TNF-α were measured. The level of IL-6 in serum was upregulated in Mod group comparing with NC group. After intervening of camel milk and silymarin, the levels of IL-6 and TNF-α were reduced significantly in serum in mice of CaM group and PC group. Camel milk and silymarin slowed down the in inflammatory response in mice with NAFLD (*p* < 0.05, Figure 1M).

### Sequencing data quality evaluation and analysis of bacterial alpha diversity and beta diversity

**FIGURE 2:**
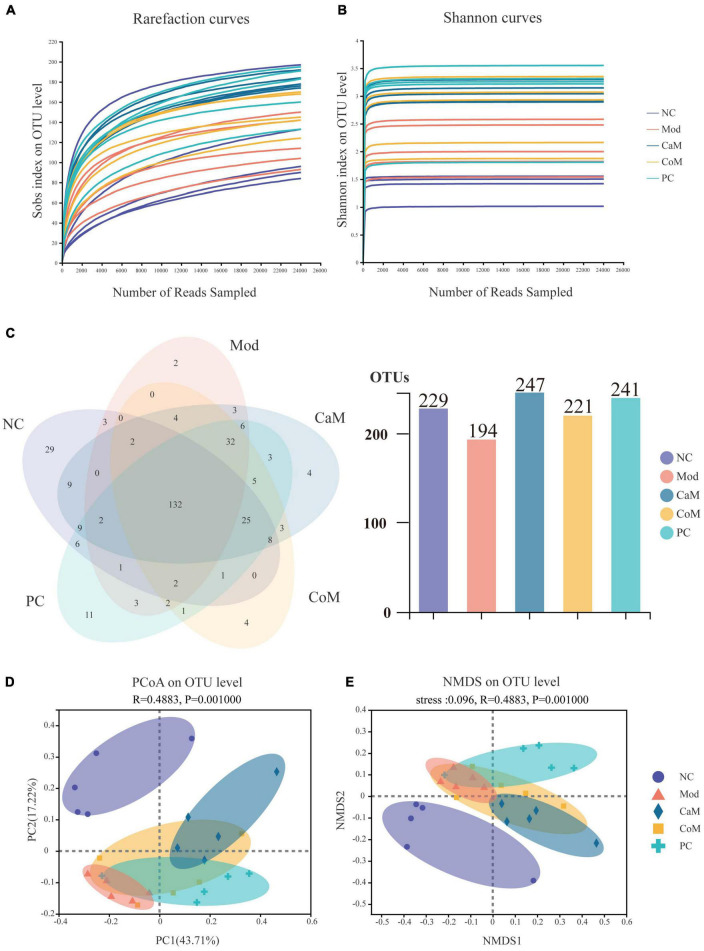
**(A)** Rarefaction curves. **(B)** Shannon curves. **(C)** Venn of operational taxonomic units (OTUs). **(D)** PCoA analysis of bacterial beta-diversity of intestinal flora. **(E)** NMDS analysis of bacterial beta-diversity of intestinal flora.

To evaluate the sequencing quality and depth of the 16S rRNA gene in the colonic contents of NAFLD mice, Sob index and Shannon index dilution curves were plotted. The Sob index curve gradually increased with the increase of sample sequencing depth and finally leveled off. And the Shannon index curve reached a plateau (Figures 2A,B). The sequencing coverage was good, and the Coverage index was higher than 0.99 (Table 6), which can truly reflect the microbial community status in the samples. The Ace and Chao indices reflect the richness of the microbial communities contained in the samples. Compared to the NC group (Table 6), there was no significant difference in the Ace index and Chao index in the Mod group (*p* > 0.05). After 12 weeks of camel milk and silymarin intervention, the Ace index and Chao index were significantly higher in the CaM group and PC group compared to the Mod group (*p* < 0.05); the Ace index and Chao index were not significantly different between the CoM and Mod groups (*p* > 0.05). In summary, camel milk and silymarin can enhance the diversity of intestinal flora Alpha in NAFLD mice.

**TABLE 6:** Alpha diversity.

Group	Alpha diversity		
	Ace	Chao	Coverage
NC	153.94 ± 33.74^c^	149.07 ± 42.56^c^	0.9988 ± 0.0003^b^
Mod	138.39 ± 23.32^c^	137.52 ± 21.35^c^	0.9991 ± 0.0002^a^
CaM	198.67 ± 4.47^a^	200.40 ± 6.95^a^	0.9989 ± 0.0002^ab^
CoM	163.15 ± 16.10^bc^	161.67 ± 16.68^bc^	0.9992 ± 0.0001^a^
PC	191.53 ± 28.47^ab^	195.63 ± 29.99^ab^	0.9989 ± 0.0003^ab^

Different superscript letters represent two data with significant differences (*p* < 0.05).

A total of 321 OTUs were detected by sequencing (Figure 2C). Long-term intake of a high-fat diet affecting the structure of the intestinal flora led to a decrease in the number of OTUs in the intestinal contents of mice, which may be due to differences in the structure of the diet. Compared with the Mod group, there were 53 more OTUs in the CaM group, 27 more OTUs in the CoM group, and 47 more OTUs in the PC group (Figure 2C).

To understand the overall differences in intestinal flora diversity between different groups, PCoA and NMDS plots were drawn based on the Brary-Curtis distance algorithm (Figures 2D,E). Samples with smaller differences in gut microbial community composition were distributed closer in the graph. As shown in Figure 2D, the samples of NC group were discrete, and the aggregation area of Mod group was smaller; however, Mod group and NC group mice were significantly separated (*p* < 0.05), indicating that the structure of intestinal flora has been changed in Mod group observably. The CaM group and the Mod group were distributed in different areas and far away from each other, indicating that the composition of the intestinal microbial community differed greatly between these two groups. The distribution areas of samples in the CoM, CaM, and PC groups partially overlapped, which may be due to the presence of the same or similar microbial communities in these three groups. However, the composition of the gut microbial community in the CoM and PC groups was significantly different from that in the Mod group (*p* < 0.05). This result infers that camel milk, cow milk, and silymarin can effectively change the structure of intestinal microbial community composition in mice. Furthermore, the results of NMDS plot analysis were highly similar to PCoA plot analysis, which further demonstrated that camel milk had a good regulatory effect on the intestinal microbial community structure of NAFLD mice.

### Analysis of the differences in the composition of intestinal flora between groups

**FIGURE 3:**
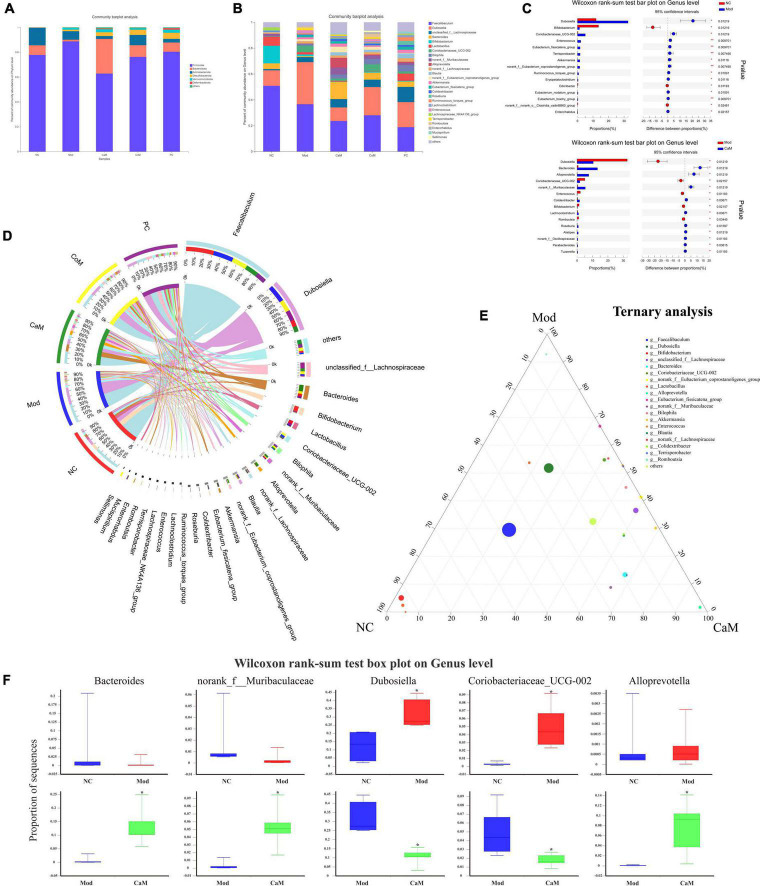
**(A)** The relative abundance of bacterial communities is at phylum level. **(B)** The relative abundance of bacterial communities is at genus level. **(C)** Relationship between groups and genus. **(D)** Comparation in NC, Mod, and CaM group. **(E)** Ternary analysis. **(F)** Wilcoxon rank-sum test box plot on genus level.

The community Bar map was drawn to understand the dominant species of each group and their proportion. As Figure 3A, the intestinal flora of the NC group had mainly Firmicutes, Bacteroidota and Actinobacteriota at the phylum level. Due to the long-term intake of high-fat diet, the Mod group showed a greater difference in the structure of the bacterial flora compared to the NC group, with an increase in the relative abundance of Firmicutes and a decrease in the relative abundance of Bacteroidota and Actinobacteriota in the Mod group. At the phylum level, the main intestinal flora dominant in the CaM group were Firmicutes and Bacteroidota, and the relative abundance of Actinobacteriota was reduced and that of Desulfobacterota was increased compared with the Mod group. The dominant intestinal flora in the CoM group of mice at the phylum level were mainly Firmicutes, Bacteroidota, and Actinobacteriota. The composition of the intestinal flora of the mice in the PC group was slightly different from that of the CoM group, in which the dominant flora at the phylum level were mainly Firmicutes, Bacteroidota, and Desulfobacterota. The Firmicutes to Bacteroidota ratio is an important basis for evaluating the structure of the flora. From Figure 3A, the Firmicutes to Bacteroidota ratio was higher in the Mod group than in the NC group (NC group: 10.01, Mod group: 66.67), and it was reduced after camel milk, cow milk, and silymarin interventions (CaM group: 2.25, CoM group: 8.10, PC group: 10.86).

At the genus level, the Mod group had altered intestinal flora structure compared to the NC group (Figures 3B,D). Figure 3C showed the genus with marked differences in relative abundance in the NC, Mod, and CaM groups. Compared to the NC group, the Mod group showed a decrease in the relative abundance of *Faecalibaculum*, *Bifidobacterium*, *Bacteroides*, *Lactobacillus*, and an increase in the relative abundance of *Dubosiella* and unclassified*_f_Lachnospiraceae*,*Coriobacteriaceae_UCG-002* (Figure 3D). After camel milk intervention, the relative abundance of *Faecalibaculum*, *Dubosiella*, *Bifidobacterium*, *Lactobacillus*, *Coriobacteriaceae_UCG-002* were decreased and the relative abundance of unclassified_f_*Lachnospiraceae*, *Bacteroides*, norank_f_*Muribaculaceae*, *Alloprevotella*, *Colidextribacter* were increased in the CaM group compared to the Mod group. In addition, the relative abundance of *Faecalibaculum*, *Dubosiella*, and *Coriobacteriaceae_UCG-002* were increased and *Bacteroides* was decreased in the CoM group compared to the CaM group. Compared with the Mod group, the relative abundance of *Faecalibaculum*, *Dubosiella* and *Coriobacteriaceae_UCG-002* were decreased, and *Bacteroides*, norank_f_*Muribaculaceae* and *Colidextribacter* were increased in the PC group. The results are consistent with the changes of CaM group. According to the ternary analysis, five important genuses were screened and intergroup comparison was done for each of these five genuses, which were *Bacteroides*, norank_f_*Muribaculaceae*, *Dubosiella*, *Coriobacteriaceae_UCG-002*, and *Alloprevotella* (Figures 3E,F).

**FIGURE 4:**
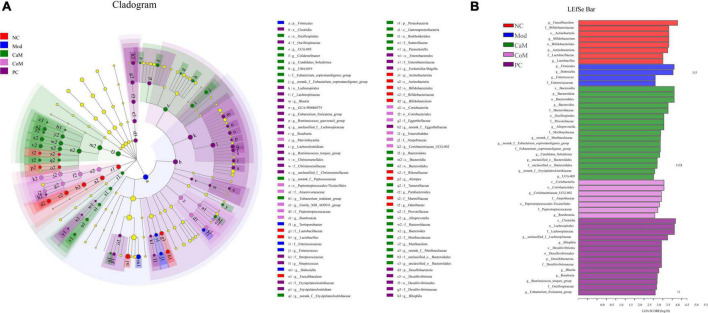
**(A)** LEfSe multi-level species hierarchy tree diagram, the circles from inner to outer in the diagram represent the phylum, class, order, family, and genus levels, each circle at different taxonomic levels represents a taxon under that level, the diameter of the circle is proportional to the relative abundance, species with significant differences follow the grouping for coloration, species without significant differences are in yellow. **(B)** Histogram of linear discriminant analysis (LDA) discrimination results, LDA value > 2.5.

To display the taxonomic differences in bacterial groups between groups, LEfSe analysis was performed and the species with statistically different bacterial diversity in relative abundance between groups were shown in the Figures 4A,B. At the genus level, *Faecalibaculum*, *Bifidobacterium* and *Lactobacillus* were main differential microbiota in NC group; *Dubosiella* and *Enterococcus* were main differential microbiota in Mod group; *Bacteroides*, *Alloprevotella*, norank_f_*Muribaculaceae*, unclassified_o_*Bacteroidales*, and *UCG-005* were main differential microbiota in CaM group; *Coriobacteriaceae_UCG-002* and *Romboutsia*, were main differential microbiota in CoM group; unclassified_f_*Lachnospiraceae*, *Bilophila*, *Blautia*, and *Roseburia* were main differential microbiota in PC group. Therefore, camel milk, cow milk, and silymarin cause alterations in the intestinal flora of NAFLD mice of significantly different species.

### The relationship between physiological indicators and genuses

**FIGURE 5:**
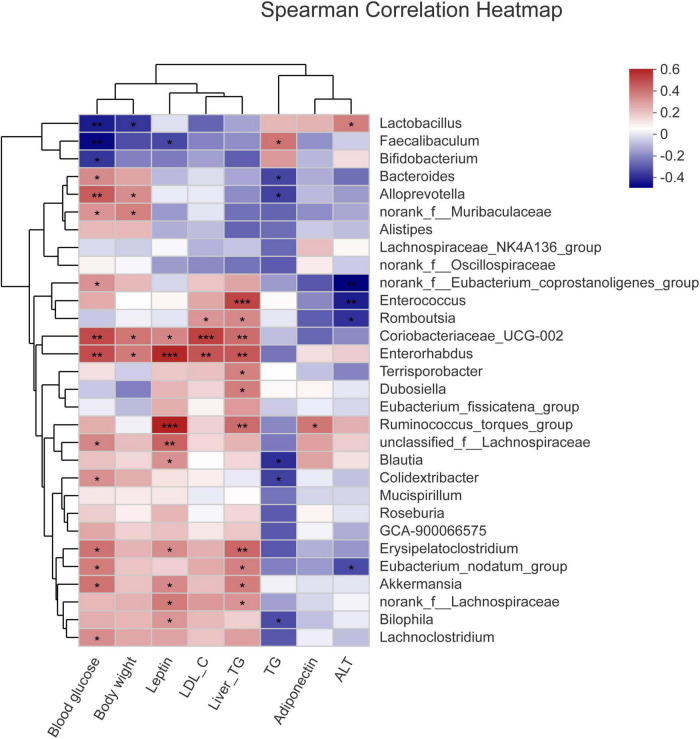
Correlation analysis between the screened physiological and biochemical indices and genuses of bacteria. *p < 0.05, **p < 0.01, ***p < 0.001.

To study the relationship between various physiological and biochemical indices and genus of mice in each group, the variance inflation factor (VIF) scores of various physiological and biochemical indices were conducted (Table 7). The indices with VIF < 10 were screened out. The correlation analysis between the screened physiological and biochemical indices and genus of bacteria was conducted (Figure 5). The results found that the relative abundance of *Dubosiella*, *Coriobacteriaceae_UCG-002* significantly and positively correlated with body weight, blood glucose, the levels of leptin, LDL-c in serum, and the levels of TG in liver. *Bacteroides*, norank_f_*Muribaculaceae*, *Alloprevotella* positively correlated with TG level in serum, and negatively correlated with TG level in liver. This result was consistent with the Section “Results.”

**TABLE 7:** VIF scores of biochemical indices.

Index	VIF value
Body weight	9.2131
Adiponectin	8.7721
ALT	7.9896
LDL-c	6.8036
Blood glucose	6.4660
Serum TG	5.9184
Leptin	5.1159
Liver TG	2.7365

### Functional prediction analysis of intestinal flora genes

**FIGURE 6:**
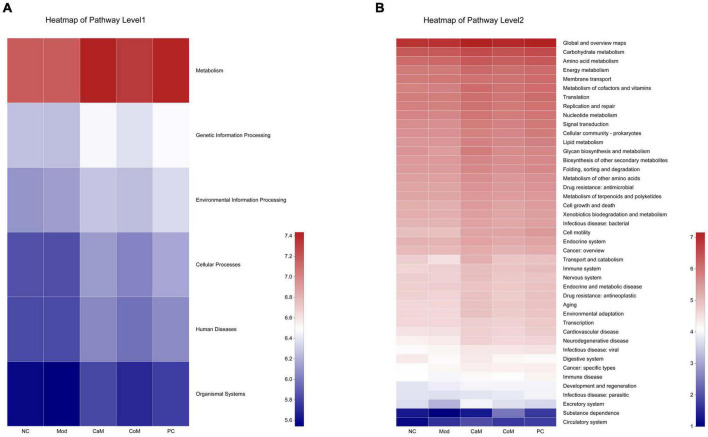
Predicting metabolic function genes based on PICRUSt2 analysis. **(A)** Heatmap of pathway level 1. **(B)** Heatmap of pathway level 2.

According to the analysis of intestinal flora diversity in NAFLD mice, long-term intake of high-fat diet changed the structure of intestinal flora in mice. The intestinal flora structure of mice was regulated after camel milk and silymarin intervention. The results of various physiological indicators and tissue sections also demonstrated that camel milk and silymarin could effectively alleviate liver lipid accumulation in mice. Identifying the KEGG metabolic pathway intestinal flora involved in combining intestinal flora data. From Figure 6A, the genes’ functional abundance of Metabolism, Genetic information processing, Environmental information processing, Cellular processes, Human diseases, Organismal systems were the same in the NC and Mod groups at the level 1. The genes’ functional abundance in the CoM group was slightly higher than Mod group. After camel milk and silymarin intervention, the intestinal flora structure of mice in the CaM and PC groups was regulated, and genes’ functional abundance was significantly higher in the CaM and PC groups than in the Mod group (*p* < 0.05). At the level 2 (Figure 6B and Table 8), there was no significant difference Carbohydrate metabolism, Amino acid metabolism, Energy metabolism, Metabolism of cofactors and vitamins, Lipid metabolism, Glycan biosynthesis and metabolism, Metabolism of terpenoids and polyketides and Xenobiotics biodegradation and metabolism in genes’ functional abundance in NC group, Mod group, and CoM group (*p* > 0.05). The genes’ functional abundance of metabolism-related increased in the CaM group and PC group after camel milk and silymarin intervention. It can be concluded that camel milk and silymarin induce dynamic changes of genes’ functional abundance related to intestinal flora metabolism in NAFLD mice, which reacted on the intestinal flora bacterial diversity.

**TABLE 8:** Predicting metabolic function genes based on PICRUSt2 analysis.

Pathway level 1	Pathway level 2
Metabolism	Carbohydrate metabolism
	Amino acid metabolism
	Energy metabolism
	Metabolism of cofactors and vitamins
	Nucleotide metabolism
	Lipid metabolism
	Glycan biosynthesis and metabolism
	Biosynthesis of other secondary metabolites
	Metabolism of other amino acids
	Metabolism of terpenoids and polyketides
	Xenobiotics biodegradation and metabolism
Genetic information processing	Translation
	Replication and repair
	Folding, sorting, and degradation
	Transcription
Environmental information processing	Signal transduction
	Environmental adaptation
Cellular processes	Cellular community–prokaryotes
	Cell growth and death
	Cell motility
	Development and regeneration
Human diseases	Endocrine and metabolic disease
	Cancer: overview
	Cardiovascular disease
	Neurodegenerative disease
	Infectious disease: viral
	Immune disease
	Cancer: specific types
	Immune disease
	Infectious disease: parasitic
Organismal systems	Endocrine system
	Immune system
	Nervous system
	Digestive system
	Digestive system
	Circulatory system

## Discussion

Non-alcoholic fatty liver disease is a chronic metabolic disease with worldwide prevalence, affecting a large number of people worldwide, characterized by obesity and excessive accumulation of lipids in the liver (1, 2). The most effective treatment for NAFLD is weight loss and enhancement of hepatic lipid metabolism. After a literature survey, we found that for those who are unable to achieve alleviation of NAFLD through lifestyle changes, bariatric surgery can also be used to reduce weight and thereby alleviate NAFLD (34). Currently, there are no drugs approved for the treatment of NAFLD specifically. Statins, pioglitazone, metformin, fenofibrate, vitamin E, and ursodeoxycholic acid are commonly used clinically to indirectly alleviate NAFLD (35–38). These drugs alleviate NAFLD through hypolipidemic effects, increased insulin sensitivity of the body, hypoglycemic effects, antioxidant effects, and enhancement of bile acid metabolism. However, some drugs had adverse effects on kidney and hepatotoxic (39–41). There are other substances that have also been shown to alleviate NAFLD. In a study by Kong et al., orange peel was used as an intervention for NAFLD and it was found that orange peel reduced body weight and lipid accumulation in the liver of animals and regulated the lipid metabolic status in animals (42). Curcumin, an extract from the rhizome of turmeric, has antioxidant and anti-inflammatory properties and was shown to reduce body mass index (BMI) and the level of TC in serum in patients with NAFLD in an investigation (43). Berberine is effective in reducing NAFLD, while some studies have claimed that its metabolite Berberrubine also has anti-obesity effects (44). Silymarin is a mixture including silybin, isosilybin, and silychristin. Mengesha et al. demonstrated that silymarin reduced hepatic steatosis and enhanced liver function in the animals (45). In addition, Ling Ren et al. used silymarin to intervene in NAFLD and found that silymarin reduce the symptoms of NAFLD by regulating the intestinal flora structure of animals (46). Therefore, in this trial, we chose silymarin as a positive drug as a reference. It is not denied that long-term medication for the treatment of NAFLD reduces the quality of life of patients (47). Therefore, it is necessary to develop health food products that effectively intervene in NAFLD.

There are more than 100 trillion microorganisms in the human gut (about 1.5 kg total weight), making it one of the most diverse ecosystems (48). It has been reported that camel milk can regulate the intestinal flora structure of mice with colitis, reduce the inflammatory response in mice, and reduce colitis in mice (49). Therefore, to study the effect of camel milk on the regulation of intestinal flora dysbiosis in NAFLD mice, we established a NAFLD mice model by feeding mice high-fat diets to change the structure of intestinal flora in mice. In the present study, as the experiment progressed, the Mod group of mice continued to increase food intake, body weight, and blood glucose. At the end of the trial period, compared with the NC group, the Mod group mice had significantly higher liver index and severe liver lipid accumulation (*p* < 0.05). And from the pathological sections, it can be observed that the Mod group mice have more lipid droplet in the liver and the liver tissue structure is severely damaged. This shows the success of the model building. Moreover, by comparing the body weight changes, blood glucose changes, liver lipid accumulation and liver tissue structure of mice in Mod and CaM groups, it can be determined that camel milk has a protective effect on the liver of NAFLD mice.

Serum biochemical indicators are important in the evaluation of NAFLD. In moderate amounts, leptin enhances hepatic TG transport, but excessive leptin increases the level of TG in the liver (50, 51). Adiponectin is a hormone secreted by adipose tissue that alleviates insulin resistance (52). High level of LDL-c in serum is an important factor in causing atherosclerosis, while high level of HDL-c in serum is an important factor in anti-atherosclerosis (53, 54). In this study, compared with the Mod group, camel milk significantly reduced the levels of leptin and LDL-c in serum and significantly increased the levels of adiponectin and HDL-c in serum in NAFLD mice (*p* < 0.05). These results, consistent with the insulin resistance index, suggest that camel milk reduced insulin resistance in NAFLD mice. In addition, camel milk reduced the inflammatory response in the NAFLD mice because of decrease of the levels of IL-6 and TNF-α in serum.

Long-term intake of high-fat diets did not change the Ace and Chao indices of intestinal flora Alpha diversity in the Mod group comparing with the NC group, suggesting that long-term intake of high-fat diets failed to change intestinal flora diversity in mice. We speculate that this may be a characteristic of the feed (which only causes NAFLD without changing the diversity of the mice’s intestinal flora). Which is different from previous studies, and the exact reason needs to be verified in subsequent supplementary experiments (55, 56). After gavage of camel milk, cow milk, and silymarin to mice, the Ace index and Chao index in the Alpha diversity of intestinal flora of mice in CaM and PC groups were significantly higher (*p* < 0.05), but the Ace index and Chao index of mice in CoM group were not significantly different from Mod group (*p* > 0.05), indicating that camel milk and silymarin could increase the number of species and the relative abundance of intestinal flora of mice. Beta diversity analysis showed that the samples of NC, Mod, CaM, CoM, and PC groups were distributed in different areas, while CoM and PC groups had some overlapping areas with Mod group; indicating that camel milk had significant effects on the intestinal flora structure of mice, while the effects of cow milk and silymarin on the intestinal flora structure of mice were different from camel milk. Combined with the analysis of physiological and biochemical indicators in mice, we can conclude that camel milk has a positive effect on the regulation of intestinal flora in NAFLD mice.

The occurrence of disease often means the change of intestinal flora, so the degree of disease occurrence and the trend of improvement can be judged by the structure of intestinal flora. At the phylum level, the ratio of Firmicutes to Bacteroidota affects the major components of the intestinal flora and is important for the assessment of metabolic disorders (57, 58). In the present study, compared with the NC group, the relative abundance of Firmicutes increased and the relative abundance of Bacteroidota decreased in the Mod group, and the ratio of Firmicutes to Bacteroidota increased. After camel milk intervention, the ratio of Firmicutes to Bacteroidota in the intestinal flora of mice in the CaM group decreased significantly (*p* < 0.05), indicating that camel milk improved the main components of the intestinal flora of NAFLD mice and had a regulation of intestinal flora diversity in NAFLD mice, thus alleviating NAFLD in mice. This change was also observed in the CoM and PC groups, but unlike the CaM group, NAFLD was not alleviated in the CoM group mice. The results of this study are similar to those of Wang and Zhao et al. (6, 59). At the genus level, *Bacteroides* is an important component of the intestinal flora and has an important impact on health. Dai et al. (60) demonstrated that *Bacteroides* was negatively correlated with obesity and type II diabetes, while Cheng’s et al. (61) results demonstrated that *Bacteroides* degrades polysaccharides and works with other genus to produce short-chain fatty acids, which are important for intestinal health at high levels in the gut; Russell et al. (62) also demonstrated that the secondary metabolites of *Bacteroides* improve the immune system. In the present study, chronic high-fat diet reduced the relative abundance of *Bacteroides* in the intestinal flora of mice in the Mod group. The relative abundance of *Bacteroides* in the intestinal flora of mice gavaged with camel milk, cow milk, and silymarin increased significantly, with the highest increase in the intestinal flora of mice in the CaM group (*p* < 0.05). Subsequent clustering analysis by genus and physiological indicators revealed that *Bacteroides* was significantly and negatively correlated with serum TG level and liver TG level (*p* < 0.05). This suggests that *Bacteroides* plays an important role in alleviating hepatic lipid accumulation in NAFLD mice. Short-chain fatty acids in the gut help protect the integrity of the intestinal barrier, and some studies have claimed that norank_f_*Muribaculaceae* is positively correlated with intestinal short-chain fatty acid levels (63, 64). In the present study, camel milk and silymarin upregulated the relative abundance of norank_f_*Muribaculaceae* in the intestine of NAFLD mice, and we also found that the relative abundance of norank_f_*Muribaculaceae* was negatively correlated with serum TG level and liver TG level in the clustering analysis of bacterial genera and physiological indicators, suggesting that norank_f_*Muribaculaceae* may regulate the levels of TG in serum and liver by increasing the content of short-chain fatty acids in the intestine. *Dubosiella* is positively associated with obesity and induces an inflammatory response in the body (65, 66). In the present study, the cluster analysis of genus and physiological indicators showed a positive correlation between *Dubosiella* and the level of TG in liver. The relative abundance of *Dubosiella* in the Mod group was significantly higher than that in the NC group (*p* < 0.05). After gavage of camel milk, cow milk, and silymarin in mice, the relative abundance of *Dubosiella* was reduced in the CaM, CoM, and PC groups, with the greatest reduction in the relative abundance of *Dubosiella* in the CaM group. This indicates that camel milk enhances hepatic TG metabolism and reduces hepatic lipid accumulation, which is consistent with the physiological index, which is also similar to the findings of Bai et al. (67). In addition, *Coriobacteriaceae_UCG-002* was found to be enriched in the Mod and CoM groups in this study, while the relative abundance of *Coriobacteriaceae_UCG-002* was significantly reduced in the CaM and PC groups compared to the Mod group. In the subsequent cluster analysis of genus and physiological indicators, *Coriobacteriaceae_UCG-002* was found to be significantly positively correlated with body weight, blood glucose, serum LDL-c level, serum leptin level, and liver TG level (*p* < 0.05). These results were consistent with physiological and biochemical parameters and pathological sections of the liver. Studies have confirmed that *Alloprevotella* has a positive effect on improving the structure of the intestinal flora and protects the integrity of the intestinal barrier (68). In the present study, it is noteworthy that *Alloprevotella* was found to be significantly enriched in the CaM group by multiple group comparisons at the genus level and heat map clustering analysis (*p* < 0.05), and a significant negative correlation was found between *Alloprevotella* and the levels of TG in serum and liver, which may be an important reason for the reduced degree of liver lipid accumulation in the CaM group of mice. *Lactobacillus* and *Bifidobacterium* are considered to be beneficial bacteria, and the relative abundance of *Faecalibaculum* is was positively correlated with health status (57, 58). However, in our study, compared to NC group the relative abundance of *Lactobacillus*, *Bifidobacterium*, and *Faecalibaculum* in Mod group, CaM group, Cow group, and PC group was reduced. The relative abundance of *Lactobacillus* and *Bifidobacterium* in the intestinal flora of mice did not increase after camel milk and cow milk interventions, which may be due to differences in diet (control diet and high-fat diet). In conclusion, camel milk, and silymarin reduced the relative abundance of harmful bacteria in the intestinal flora of NAFLD mice, improved the structure of the intestinal flora and protected the integrity of the gut barrier. Although cow milk also changed the structure of intestinal flora in NAFLD mice, it did not effectively prevent lipid accumulation in the liver of mice.

Carbohydrate, amino acid, and lipid, as the three basic nutrients of the body, are the basis for the normal physiological activities of the body (69, 70). Cofactors and vitamins play a crucial role in the metabolic activities of carbohydrate, amino acid, and lipid (71–73). Meanwhile, the energy required for the metabolic activities of carbohydrate, amino acid, and lipid is provided by the mitochondria (the main site of energy metabolism), and mitochondrial dysfunction affects the normal metabolic processes of the body (74). In the present study, Carbohydrate metabolism, Amino acid metabolism, Energy metabolism, Metabolism of cofactors and vitamins and Lipid metabolism were observed to be significantly enhanced in NAFLD mice after camel milk and silymarin intervention. It means that camel milk and silymarin enhanced the energy metabolism of NAFLD mice, which in turn promoted the metabolism of the three major nutrients and further slowed down the accumulation of lipids in the liver. However, based on the 16S rRNA gene prediction is the basic metabolic function of microorganisms, it is necessary to design experiments to verify the effect of camel milk on NAFLD mice in terms of transcription level, protein level and phenotype regarding metabolism from the test animals themselves.

Conclusively, camel milk improved various physiological and biochemical indicators in NAFLD mice, positively regulated the structure of intestinal flora and modulate metabolism-related functional genes. Therefore, the results of this study provide a basis for camel milk to alleviate NAFLD.

## Conclusion

Non-alcoholic fatty liver disease is a metabolic disorder with a wide range of effects, most notably characterized by the excessive accumulation of lipids in the liver. Interventions for NAFLD are particularly important in the absence of drugs specifically for the treatment of NAFLD. Our study demonstrates that camel milk reduced lipid accumulation in the liver, maintained normal liver tissue structure, enhanced hepatic glycolipid metabolism, and reduced inflammatory response in NAFLD mice. The analysis of intestinal flora of NAFLD mice demonstrated that camel milk regulated the intestinal flora structure of mice, increased intestinal flora diversity, reduced the relative abundance of *Dubosiella* and *Coriobacteriaceae_UCG-002*, which were positively correlated with liver TG level, and increased the relative abundance of *Bacteroides*, norank_f_*Muribaculaceae*, and *Alloprevotella*, which were negatively correlated with liver TG level. In addition, camel milk may also enhance the metabolism of the three major nutrients in the organism of NAFLD mice. Therefore, camel milk has a palliative effect on NAFLD in mice.

## Data availability statement

The datasets presented in this study can be found in online repositories. The names of the repository/repositories and accession number(s) can be found below: https://www.ncbi.nlm.nih.gov/, PRJNA889750.

## Ethics statement

The animal study was reviewed and approved by the Key Laboratory of Dairy Biotechnology and Engineering, Ministry of Education, Inner Mongolia Agricultural University, Hohhot, China.

## Author contributions

SH, LM, and YL designed the experiments. SH, YL, HL, and LL conducted most of the experiments. SH wrote and edited the manuscript. TJ and RJ reviewed the manuscript. All authors read and approved the manuscript.
